# Accuracy and cutoff values of delayed heart to mediastinum ratio with ^123^I-metaiodobenzylguanidine cardiac scintigraphy for Lewy body disease diagnoses

**DOI:** 10.1186/s12883-015-0338-9

**Published:** 2015-05-15

**Authors:** Guillaume Lamotte, Rémy Morello, Adrien Lebasnier, Denis Agostini, Gilles L. Defer

**Affiliations:** Department of Neurology, University Hospital of Caen, Avenue Côte de Nacre, 14033, Caen, Basse Normandie France; Unité INSERM U 919, Sérine protéase et physiopathologie de l’Unité Neurovasculaire, GIP Cycéron, Université Caen Basse-Normandie, Caen, France; Department of Statistics and Clinical Research, University Hospital of Caen, Caen, France; Department of Nuclear Medicine, University Hospital of Caen, Caen, EA 4650, France

**Keywords:** Sympathetic innervation, Parkinson's disease, parkinsonism, MIBG, Myocardium, Lewy body dementia, Diagnosis

## Abstract

**Background:**

Different studies have found diminished cardiac metaiodobenzylguanidine (MIBG) uptake in Lewy body (LB) related conditions (Parkinson’s disease (PD) and Lewy body dementia (LBD)). However, delayed heart/mediastinum (d-H/M) ratio diagnostic cutoff points are debated in parkinsonian syndromes.

**Methods:**

We performed a monocentric retrospective analysis on 62 consecutive parkinsonian patients who underwent an ^123^I-MIBG scintigraphy, brain imaging and dopaminergic imaging using ^123^I-Ioflupane single photon emission computed tomography (SPECT) from 2009 to 2013. The optimal d-H/M ratio was determined from a Receiver Operating Characteristic (ROC) curve and the sensitivity (Se), specificity (Sp) and likelihood ratios (LR) were calculated. 42 patients were diagnosed with LB diseases (20 PD, 22 LBD) and 20 patients with other diseases.

**Results:**

^123^I-MIBG scintigraphy helped to distinguish PD (p < 0.001) and LBD (p = 0.03) from other diseases. The optimal d-H/M ratio was 1.48 (0.85 area under the ROC curve). Se and Sp were 83.3 %, and 85 % respectively with positive and negative LR of 5.5 and 0.2 respectively. Patients with LBD had a lower d-H/M ratio than patients with PD (result not statistically significant) and a cutoff point at 1.2 could help to differentiate the two diseases. We did not find any correlation between the d-H/M ratio and clinical or ^123^I-Ioflupane SPECT data.

**Conclusion:**

According to our population, the d-H/M ratio at 1.48 led to the best performance diagnosis with good Se, Sp and accuracy. In addition, a d-H/M ratio cutoff at 1.2 could help to differentiate PD from LBD.

## Background

**Fig. 1:**
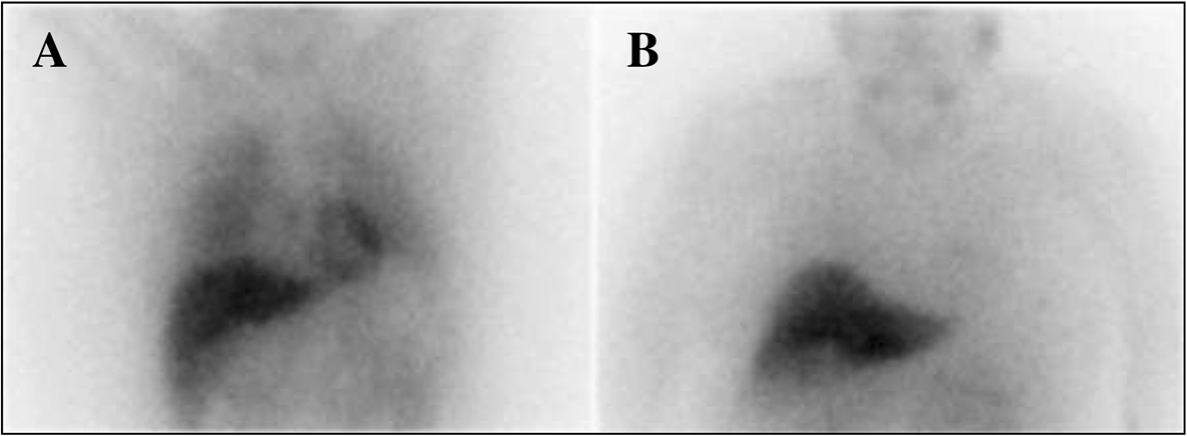
Planar 123-I MIBG cardiac scintigraphy images. **a**. ^123^MIBG cardiac scintigraphy from a patient with multiple system atrophy with normal cardiac MIBG uptake in delayed image. **b**. ^123^MIBG cardiac scintigraphy from a patient with Parkinson’s disease with marked reduction of cardiac MIBG uptake in delayed image

Supporting clinical diagnosis of a parkinsonian syndrome can be challenging, particularly during the initial phase of the disease. However, improvement of diagnostic accuracy may be important as it influences patient management, especially if neuroprotective strategies are developed in the near future. Metaiodobenzylguanidine (MIBG) is a false adrenergic neurotransmitter analogue of norepinephrine. ^123^I-MIBG cardiac scintigraphy is a diagnostic technique to evaluate cardiac sympathetic innervation and noradrenergic post-ganglionic cardiac denervation, a common feature in Parkinson’s disease (PD) and Lewy body dementia (LBD) [1–5]. It has been suggested that ^123^I-MIBG cardiac scintigraphy could help to distinguish parkinsonian syndromes and three recent meta-analyses suggested very good Se and Sp with this technique to differentiate Lewy body (LB) diseases from other neurodegenerative forms of parkinsonism (Fig. 1) [6–8]. However, there is great variability in technical aspects of the procedure between centers and a proposal for standardizing the technique and the interpretation of the images has been published recently [9]. The delayed heart/mediastinum ratio (d-H/M) is calculated as the ratio of the count density for the left ventricle to that for the upper mediastinum from anterior planar images. This ratio provides information regarding the uptake, storage and release of MIBG at nerve endings and is widely used as a quantitative index of cardiac ^123^I-MIBG uptake [9]. The aim of this study was to evaluate the accuracy of the delayed heart/mediastinum (d-H/M) ratio of ^123^I-MIBG myocardial scintigraphy that we established in a population of 62 consecutive parkinsonian patients to separate LB diseases from other diagnoses.

## Methods

We performed a monocentric retrospective analysis on 62 consecutive parkinsonian patients who underwent an ^123^I-MIBG scintigraphy, brain imaging and dopaminergic imaging using ^123^I-ioflupane SPECT from January 2009 to March 2013. Evaluations were performed in the same center outside of a research purpose in order to help the clinician in patient management. The study was approved by the Institutional Ethical Committee (North West III People’s Protection Committee, University Hospital of Caen, Caen, France). All patients were originally diagnosed by a movement disorder specialist between January 2009 and March 2013. In March 2013, we verified that each patient also fulfilled the current international diagnosis criteria for each of the final clinical diagnoses (United Kingdom Parkinson’s disease Brain Bank Criteria for PD [10], revised McKeith criteria for LBD [11], criteria from Gilman et al. in 2008 for multiple system atrophy (MSA) [12], NINDS-SPSP criteria for progressive supranuclear palsy (PSP) [13] and criteria proposed by Armstrong et al. in 2013 for cortico-basal degeneration (CBD) [14]). We collected clinical data including a detailed battery of neuropsychological tests when available. Global cognition was assessed with the Mini Mental State Examination test (n = 50). Executive functions were assessed with the TMT-A test time (n = 35), the category fluency test (animals) (n = 41), the phonemic fluency test (p) (n = 36), and the Grober and Buschke free recall total score (n = 43). Memory function was assessed with the Grober and Buschke free and cued recall total scores (n = 43), the Rey complex figure delayed recall (n = 41), the digit span forward (n = 37), and the digit span backward (n = 37). Instrumental functions were assessed with praxis tests (n = 35) and the Rey complex figure copy (n = 41). When applicable, the Hoehn and Yahr (H&Y) scale was used to assess disease severity. In our population, the early stages of the disease were defined by H&Y stages 1 or 2 or less than 3 years of evolution of the disease. Patients were divided into four groups regarding the clinical presentation: akinetic-rigid (AR), tremor-dominant (T), akinetic-rigid and tremor (ART), and patients with cognitive complaints with no PD motor core symptoms. We also looked for treatments that could interfere with the uptake and/or vesicular storage of MIBG and presence of diabetes as ^123^I-MIBG myocardial uptake may be affected by various drugs and cardiac autonomic neuropathy [15, 16].

For all patients ^123^I-MIBG cardiac scintigraphy was performed according to the same protocol, which was in line with the latest recommendations from the European Cardiovascular Committee [9]. Myocardial infarction was excluded by rest gated ^201^Thallium myocardial perfusion SPECT and all patients had a normal left ventricular ejection fraction >50 %. At 15 min (early) and 4 h (delayed) after the injection of 370 MBq (10 mCi) ^123^I-MIBG, anterior planar images were obtained using a dual-head gamma camera system (SymbiaT2, Siemens) equipped with a low-energy high-resolution collimator. A static image was obtained for fixed time (10 min) with the same acquisition for all patients with a 128×128 matrix. The regions of interest (ROI) were manually drawn around the heart and the mediastinum. To define the mediastinum, the landmarks were the apexes of the lungs and the ROI were drawn on the middle of the two apexes. The ROIs’ statistics for ratios were provided from total counts. The tracer uptake was measured within each region of interest to calculate the d-H/M ratio. We did a post-hoc analysis after exclusion of patients with treatments that could interfere with the uptake and/or vesicular storage of MIBG and patients with diabetes [15]. We also collected ^123^I-ioflupane SPECT data when available (qualitative interpretation and semi quantitative analysis).

The sensitivity (Se), specificity (Sp), positive (PPV) and negative (NPV) predictive values and LR were calculated. The optimal d-H/M ratio was determined from a ROC curve. All estimates were calculated with 95 % confidence intervals (^95 %^IC). Comparisons between groups were assessed with the Fisher’s exact test. The ANOVA model (or Mann–Whitney test if the conditions of validity are not satisfied) was used to compare the means of quantitative variables in two independent groups. The relationships between the d-H/M ratio and the clinical, neuropsychological and ^123^I-ioflupane SPECT data were analyzed using the Pearson correlation coefficients. All the tests were two-tailed and their level of significance (p) was defined as p < 0.05. IBM®-SPSS® 20.0 for Windows was the statistical software used.

## Results

42 patients were diagnosed with LB diseases (20 PD, 22 LBD) and 20 patients with other diseases (MSA n = 6, PSP n = 5, CBD n = 1, drug-induced parkinsonism (DIP) n = 2, Alzheimer disease (AD) n = 2, Perry syndrome n = 1, others n = 3). Among the group “others”, 1 patient had a parkinsonian syndrome but was lost to follow-up and 2 patients came to the clinic with mnesic complaints with no PD motor core symptoms but did not meet the criteria for AD or LBD. There were no differences in demographic characteristics between LB diseases and others diagnoses. The mean follow-up time was 66 months and the mean delay between onset of symptoms and ^123^I-MIBG cardiac scintigraphy was 47.3 months. Table 1 summarizes patient characteristics.

**Table 1:** Patient characteristics

	All patients	PD	LBD	MSA	PSP	CBD	Others
N	62	20	22	6	5	1	8
Male/female	25/37	10/10	8/14	2/4	3/2	0/1	2/6
Age at diagnosis (y), mean +/− SD	64 +/− 13.4	56.7 +/− 16.2	69.8 +/− 9.3	60.7 +/− 4.5	64 +/− 9.7	70	68.2 +/− 14.9
Disease duration (mo), mean +/− SD	66 +/− 45	81.6 +/− 59.8	53.7 +/− 33.1	66.5 +/− 15.8	58.4 +/− 60.2	38	68.7 +/− 32.3
Duration of symptoms before scintigraphy (mo), mean +/− SD	47.3 +/− 43.4	65.8 +/− 59.9	35.3 +/− 25.7	47.7 +/− 19.5	48.8 +/− 57	28	35.3 +/− 29.7
AR/T/ART/Non motor	32/5/22/3	6/3/11/0	12/1/8/1	6/0/0/0	3/0/2/0	1/0/0/0	4/1/1/2
H&Y, mean +/− SD	2.4 +/− 1.2	2.4 +/− 1.2	2.3 +/− 1.1	3.6 +/− 1.2	3 +/− 0	4	1 +/− 1.1
L-Dopa-equivalent dose, mean +/− SD, (N)	407.5 +/− 436.9 (37)	520 +/− 438.2 (16)	260 +/− 374.6 (10)	638.3 +/− 502.4 (5)	840 +/− 378.2 (4)	0 (0)	139.5 +/− 262.9 (2)
DaTSCAN, N	40	11	14	5	3	1	6
Neuropsychological assessment, N	50	12	21	4	4	1	8

PD, Parkinson’s disease; LBD, Lewy body dementia; MSA, multiple system atrophy; PSP, progressive supranuclear palsy; CBD, cortico-basal degeneration; AR, akinetic-rigid; T, tremor-dominant; ART, akinetic-rigid and tremor; Non motor, cognitive complaints with no PD motor core symptoms. H&Y, Hoehn and Yahr scale

**Fig. 2:**
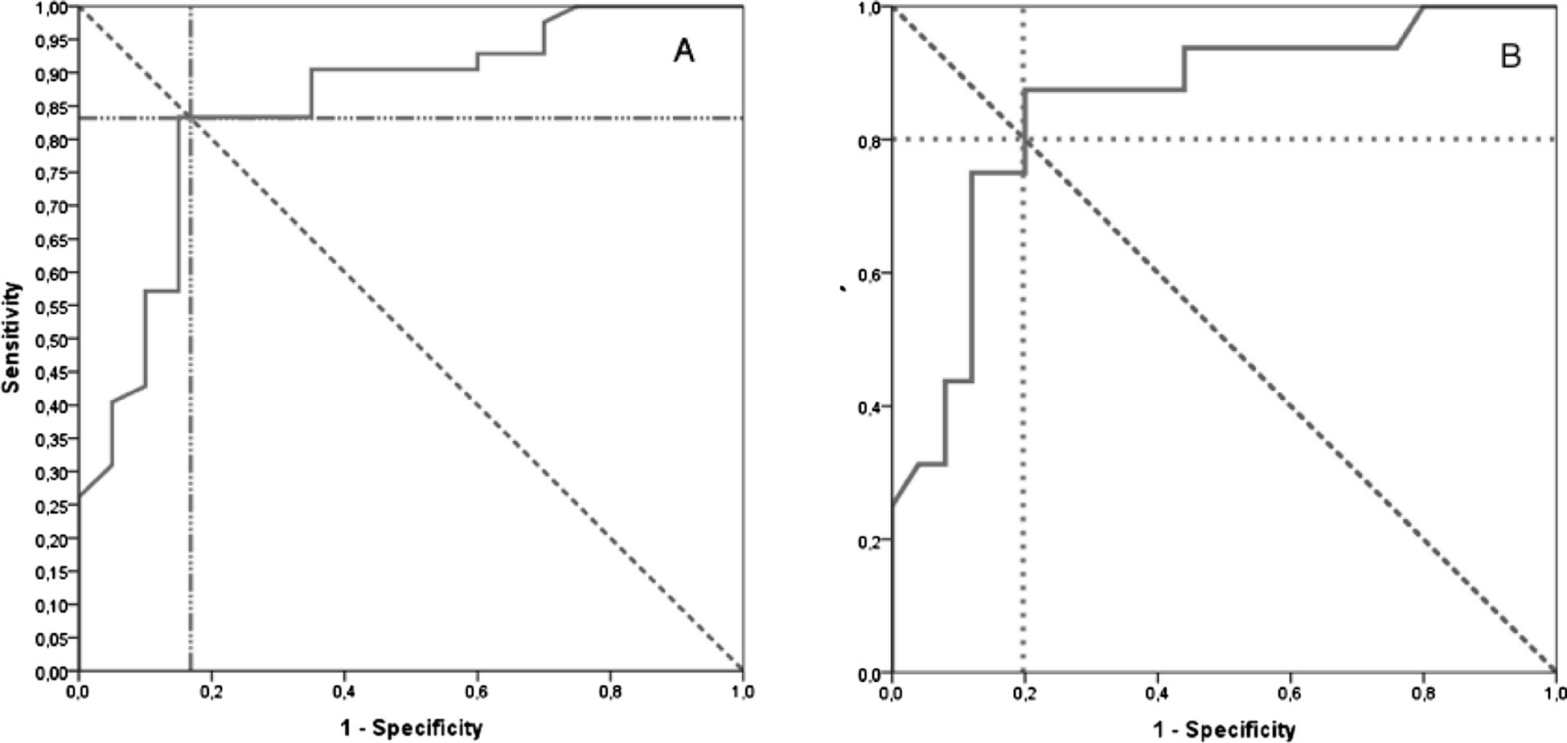
ROC curves for the differential diagnosis between LB related diseases and other diagnoses using the delayed heart to mediastinum ratio. **a**. ROC curve including the 62 patients. **b**. ROC curve after exclusion of patients with treatments that could interfere with the uptake and/or vesicular storage of MIBG and/or patients with diabetes

^123^I-MIBG scintigraphy helped to distinguish PD (p < 0.001) and LBD (p < 0.001) from other diseases. As expected, this technique did not differentiate PD from LBD (p = 0.7) (Table 2). In our population, the optimal d-H/M ratio was set at 1.48 with 0.85 area under the ROC curve (^95 %^IC 0.745-0.953; p < 0.001) (Fig. 2). Se and Sp were 83.3 %, and 85 % respectively, PPV and NPV were 92.1 % and 70.8 % respectively, and positive and negative LR of 5.5 and 0.2 respectively.

**Table 2:** Results of ^123^I-MIBG cardiac scintigraphy with a d-H/M cutoff at 1.48

	d-H/M < 1.48	d-H/M > 1.48	P-value*	P-value**
Others	3	17	<0.001	
PD	16	4		0.691
LBD	19	3	<0.001	
Others	3	17		

*Comparison between LB diseases (PD or LBD) and others

**Comparison between PD and LBD

Fisher exact test

After exclusion of patients with treatments that could interfere with the uptake and/or vesicular storage of MIBG and patients with diabetes (5 PD, 12 LBD, 2 MSA, 1 PSP, 1 other), the results were still statistically significant and ^123^I-MIBG scintigraphy helped to distinguish PD (p < 0.001) and LBD (p < 0.05) from other diseases. The optimal d-H/M ratio after exclusion of these patients was 1.48 with 0.85 area under the ROC curve (^95 %^IC 0.725-0.975; p < 0.001) and diagnostic performances were similar with Se and Sp of 87.5 % and 80 % respectively (Fig. 2). There were no significant differences between these patients and patients without any potential pharmacological interaction or without diabetes regarding the clinical presentation and the ^123^I-MIBG cardiac scintigraphy results. Mean d-H/M ratio was 1.27 +/− 0.21 for the 12 LBD patients who were excluded and 1.25+/−0.24 for the 10 other LBD patients (p > 0.05). Mean d-H/M ratio was 1.23+/−0.24 for the 5 PD patients who were excluded and 1.37+/−0.17 for the 15 other PD patients (p > 0.05).

**Fig. 3:**
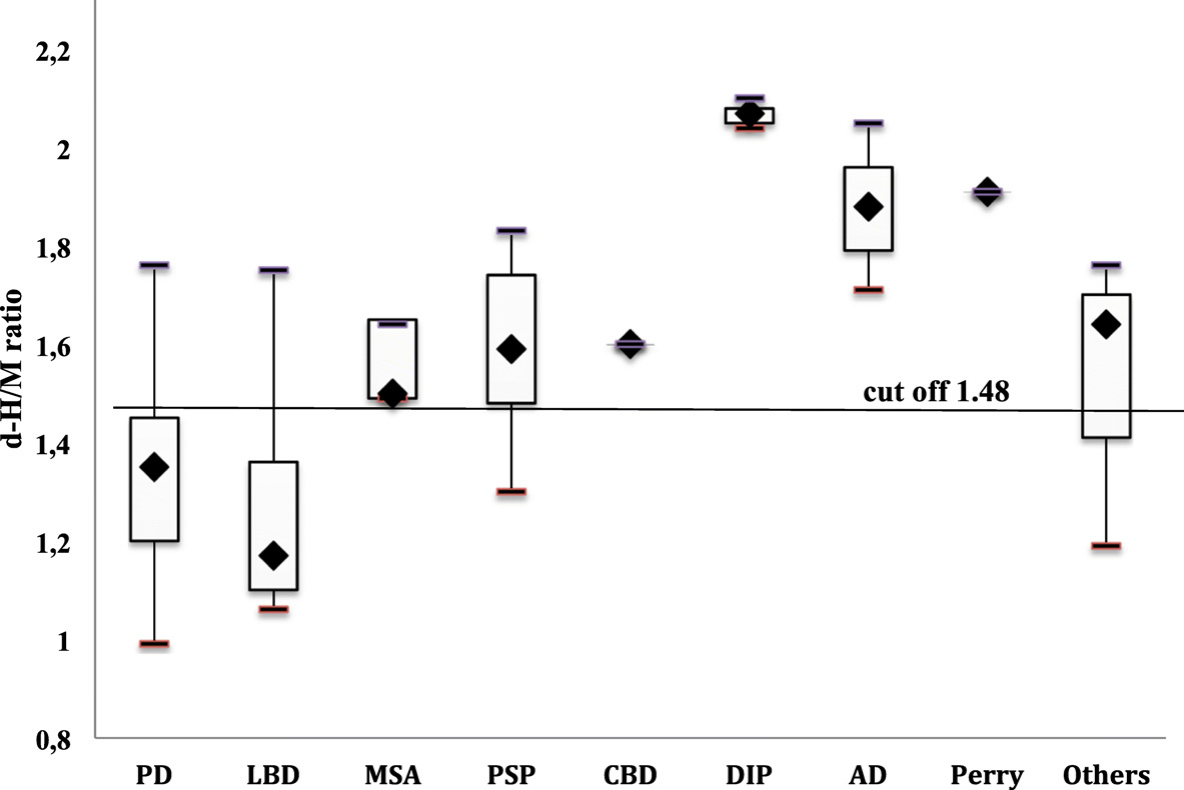
Delayed Heart to mediastinum (d-H/M) ratio of disorders. The box plots show the median values (mark in the box), 25th percentile (lower line of box), 75th percentile (upper line of box), minimum value (lower T bar), and maximum value (upper T bar) in each group. The cutoff line is set at 1.48. PD, Parkinson disease; LBD, Lewy body dementia; MSA, multiple system atrophy; PSP, progressive supranuclear palsy; CBD, cortico-basal degeneration; DIP, Drug-induced parkinsonism; AD, Alzheimer disease; Perry, Perry syndrome; Others

Fig. 3 shows the distribution of the d-H/M ratio by diagnoses. Although this was not statistically significant, patients with LBD had a lower d-H/M ratio than patients with PD. The mean value of the d-H/M ratio was 1.35 (range: 0.99-1.76) in patients with PD and 1.17 (range: 1.06-1.75) in those with LBD. A cutoff point at 1.2 for the d-H/M ratio could help to differentiate the two diseases. With this cutoff point 80 % of PD patients had a d-H/M ratio >1.20 and 80 % of patients with a d-H/M ratio <1.20 had LBD.

We did not find any significant correlation between the d-H/M ratio and clinical data including age, disease duration, disease severity, clinical phenotype and neuropsychological evaluation. We found no significant correlation between the d-H/M ratio and ^123^I-ioflupane SPECT data.

## Discussion

Cardiac sympathetic innervation was significantly impaired in LB related disorders (PD and LBD) in comparison to other diagnoses. In our population, the optimal d-H/M ratio was 1.48 and led to the best performance diagnosis with good Se, Sp and accuracy. Three recent systematic meta-analyses have suggested high performance diagnosis with this technique to differentiate LB disorders from other forms of parkinsonism with overall Se from 88 to 94 % and overall Sp from 82.6 to 91 % [6–8]. Our study suggests similar results. In addition, the d-H/M ratio at 1.48 is close to 1.43, which was the best cutoff point defined by a study by Muxi et al. comparing patients in a Caucasian population with PD with normal age- and sex-matched subjects [17]. Muxi et al. used the same type of camera and collimator (Siemens dual-head camera equipped with a low-energy high-resolution parallel-hole collimator) as our study, which supports a good interobserver reproducibility for d-H/M ratio.

Patients with LBD had a lower d-H/M ratio than patients with PD (result not statistically significant) and a cutoff point of 1.2 could help to differentiate the two diseases. Suzuki et al. have compared sympathetic myocardial innervation between LBD and PD and have reported a lower ^123^I-MIBG uptake in LBD than in controls or PD patients [18]. According to the authors, this result could reflect a greater postganglionic cardiac sympathetic denervation in LBD and/or a more important LB pathology in sympathetic ganglia in LBD compared to PD. Another hypothesis is the occurrence of transsynaptic denervation in LBD due to a greater number and wider distribution of LB in the brain of LBD patients, especially in the central autonomic control system [18]. Although the difference was not statistically significant in our population, our preliminary data support the finding that LBD patients could have a lower d-H/M ratio than PD patients, independent of disease severity. Additional studies are needed to confirm such a d-H/M ratio difference and understand the underlying pathophysiology.

Several studies found correlations between myocardial sympathetic denervation and clinical presentation in PD. Spiegel et al. and Chung et al. found a significant correlation between myocardial sympathetic denervation and severity of hypokinesia and rigidity but not with tremor [19, 20]. However, Chiaravalloti et al. found a correlation only with tremor [21]. Kim et al. have shown that cognitive decline in patients with PD was associated with a greater impairment in myocardial sympathetic denervation [22]. We did not find any correlation between the d-H/M ratio and clinical or neuropsychological data. The former could be explained by the heterogeneity and the low number of patients in our study, and the latter by the difference in the cognitive tests used and the definition of cognitive impairment between the studies.

We did not find a relationship between cerebral and cardiac changes in our PD patients, which supports the results observed by Raffel et al. in a PET study [23]. However, in a large homogeneous cohort of PD patients, Spiegel et al. found a strong correlation between cerebral nigrostriatal dopaminergic degeneration using ^123^I-ioflupane SPECT and myocardial sympathetic denervation [24]. Despite similar imaging techniques, the size difference between the two cohorts and heterogeneity of our PD group could explain the difference in the results between the two studies. Additional studies considering disease duration and disease severity are needed to answer this question.

Some studies have suggested that cardiac ^123^I-MIBG cardiac scintigraphy could be of limited value in the diagnosis of LB related disorders in the early stages of the disease because of a lower Se [25, 26]. In patients with a PD diagnosis (clinical assessment and dopamine PET evaluation), Ishibashi et al. found a Se of 53.8 % for the d-H/M ratio in the H&Y 1 and 2 PD patients [25], whereas Sawada et al. found a Se of 73.3 % for the d-H/M ratio in the subgroup with duration of the disease of ≤3 years [26]. However, in a meta-analysis Orimo et al. found a pooled Se and Sp of the d-H/M ratio at 94.1 % and 80.2 %, respectively when PD was limited to early stages (H&Y 1 and 2) [8]. In our population, there was no significant difference between early and late stages of the disease and the accuracy of ^123^I-MIBG cardiac scintigraphy remained the same between the two stages suggesting that ^123^I-MIBG cardiac scintigraphy may be yet useful as a diagnostic tool at the early phase of PD.

The main limitations of our study were the heterogeneity of our population with only 20 patients with PD, the relatively low number of subjects and the absence of histopathologically confirmed diagnoses (except Perry syndrome). However, all 62 consecutive patients were seen in a university hospital setting and all files reviewed by a movement disorder specialist and a nuclear cardiologist involved in cardiac MIBG imaging. In addition, final diagnoses were made in agreement with international diagnosis criteria and the lengthy follow-up period (66 months) would improved clinical diagnosis accuracy. The heterogeneity of our population, which includes 9 different diseases, each with a different pathophysiology, prevents us from making any definitive conclusions regarding the role of cardiac ^123^I-MIBG scintigraphy in each of these diseases individually. However, cardiac ^123^I-MIBG scintigraphy clearly detected two diagnostic clusters with good performance diagnosis: (1) LB related disorders (PD and LBD); and (2) atypical parkinsonisms without LB (MSA, PSP, DCB, Perry syndrome), and other forms of dementia such as AD. Thus, cardiac ^123^I-MIBG scintigraphy is a potential diagnostic tool for the clinician that can accurately distinguish between PD and atypical parkinsonism, and between LBD and other forms of dementia. Due to the retrospective design of the study, we were not able to verify that potential treatments that could interfere with the uptake and/or vesicular storage of MIBG were stopped before the ^123^I-MIBG cardiac scintigraphy. Despite we can suspect that some of these drugs have decreased d-H/M ratio in some of our patients, we can assume that this potential reduction was non-significant as the post-hoc analysis we performed after exclusion of these patients confirmed our initial result with an optimal d-H/M ratio at 1.48.

## Conclusion

In this study we have shown, as previously suggested, that a d-H/M ratio at 1.48 for ^123^I-MIBG cardiac scintigraphy led to the best performance diagnosis to differentiate LB related disorders (PD and LBD) from other diagnoses. In addition, a d-H/M ratio cutoff at 1.2 could help to differentiate PD from LBD. Further prospective studies in a large number of subjects with parkinsonian syndromes are needed to confirm our findings and to help to understand the pathophysiology of LB related disorders.

## Abbreviations

AD: Alzheimer disease
AR: Akinetic-rigid
ART: Akinetic-rigid and tremor
CBD: Cortico-basal degeneration
d-H/M ratio: Delayed heart/mediastinum ratio
DIP: Drug-induced parkinsonism
H&Y: Hoehn and Yahr
LB: Lewy body
LBD: Lewy body dementia
LR: Likelihood ratios
MIBG: Metaiodobenzylguanidine
MSA: Multiple system atrophy
NPV: Negative predictive value
PD: Parkinson’s disease
PPV: Positive predictive value
PSP: Progressive supranuclear palsy
ROC Curve: Receiver Operating Characteristic curve
ROI: Regions of interest
Se: Sensitivity
Sp: Specificity
SPECT: Single photon emission computed tomography
T: Tremor-dominant
